# Manufacturing Process, Tensile-Compressive, and Impact Properties of Tungsten (W)-Particle-Reinforced SLA Methacrylate

**DOI:** 10.3390/polym15244728

**Published:** 2023-12-17

**Authors:** Mattia Perin, Luca Quagliato, Guido A. Berti, Changsoon Jang, Sewon Jang, Taeyong Lee

**Affiliations:** 1 Department of Management and Engineering, University of Padua, 36100 Vicenza, Italy; mattia.perin@phd.unipd.it (M.P.); guido.berti@unipd.it (G.A.B.); 2 Division of Mechanical and Biomedical Engineering, Ewha Womans University, Seoul 03760, Republic of Korea; lucaq@ewha.ac.kr; 3 Graduate Program in System Health Science and Engineering, Division of Mechanical and Biomedical Engineering, Ewha Womans University, Seoul 03760, Republic of Korea; csoon@ewha.ac.kr (C.J.); sw9148@ewha.ac.kr (S.J.)

**Keywords:** additive manufacturing (AM), stereolithography (SLA), particle-reinforced composite, quasi-static properties, impact strength

## Abstract

The interest in research and development for additive manufacturing (AM) processes has grown significantly over the last years and attracts both industry and academia alike. Among the available AM technologies, stereolithography (SLA) is one of the most discussed, researched, and employed. On the other hand, being based on thermoset resins, all the limitations of this typology of materials still apply, limiting the range of applications of this highly versatile process. To overcome these limitations, especially brittleness, this research analyzes the effects of Tungsten (W) micro-size (average size 1 μm) particles reinforcement on a methacrylate base material. First, the manufacturing process for creating the W-reinforced methacrylate material is presented and investigated to define the effect of pre- and post-processing operations on the quality of the pre-cured solution considering 4% and 10% wt. W particles concentrations. Afterward, tensile, compressive, and impact specimens were manufactured with both concentrations and compared with the experimental results from clear (unfilled) resin-based specimens used as the benchmark. The addition of tungsten particles showed a strong improvement in the impact strength of the methacrylate base material, quantified in 28% for the 4% and 55% for the 10% wt., respectively, although at the expense of a slight reduction in elastic and yield properties on average −12%. Furthermore, using Scanning Electron Microscope (SEM) analyses, the particle–matrix interaction was investigated, showing the interaction between the polymer matrix and the reinforcement and the mechanism by which the impact resistance is enhanced.

## 1. Introduction

Among production processes, additive manufacturing solutions offer several advantages, particularly fast production from a CAD design file [1,2] to the final product and the possibility of producing near-net-shape complex geometries without additional operations while avoiding material waste [2]. Additive manufacturing includes different technologies and diversifies from polymer, ceramics, and metallic materials [3,4,5]. Therefore, it is unsurprising that AM technology is one of the greatest growing manufacturing solutions in the last decades for different industrial fields [6,7,8,9,10]. Despite several advantages, one of the weak points of AM is the relatively poor mechanical properties of the final products [11] compared to other materials used in industries, especially metals. This is the case of additive manufacturing processes involving polymeric materials [12], where the lack of relatively high stiffness and static resistance [13,14] reduce the applicability of these solutions.

To this aim, the mechanical properties of stereolithography products have been investigated by several academic research [13,14] and industrial case studies [15]. Furthermore, the post-cure step is a widely used process to modify and improve the material behavior after the printing phase. This latter process is meant to avoid any uncured resin inside and enhance the stiffness and strength of the final component [16]. Post-curing consists of a time-controlled process where the printed part is placed in a temperature-controlled chamber irradiated by ultraviolet (UV) light for a user-defined period to complete the polymerization process. To this aim, Quagliato et al. [17] investigated the correlation between different post-curing settings, specimen thicknesses, and load tests to analyze the mechanical response and the post-curing treatment uniformity across the specimen section. In addition, Kim et al. [18] analyze the application of a handheld light-emitting diode (LED) dental light-curing unit (LCU) to post-cure resin specimens, finding a potential alternative to the classic desktop post-curing system.

Coming to the focus of this contribution, it is well known that adding reinforcement particles or fibers affects the material’s mechanical response and is largely employed for both thermoset and thermoplastic polymers [19]. Recently, Almushaikeh et al. [20] proposed a review of the manufacturing systems of carbon fiber-reinforced thermoplastics products, focusing also on the carbon fiber material recycling techniques.

In this case, the fibers’ alignment to the external loads significantly affects the final component performances [21,22,23]. On the other hand, particle-filled composites normally show a slight drop in strength with an increase in impact resistance [24,25]. Moreover, adding reinforcement particles [26,27,28] to fiber-reinforced composites, especially polymer-based composites, showed to have a positive effect on both mechanical and thermal properties. In the same way, to improve the mechanical behavior of the AM-manufactured parts, different types of reinforcements on the base thermoset photopolymer have been analyzed in the literature to create high-performance composites while keeping the aforementioned production advantages [10,29]. Zhang et al. [30] investigated the influence of wood flour with different concentrations on resin components produced by SLA, reporting improved tensile strength and Young’s modulus. A combination of the SLA process with selective electroless metallization has been implemented by Credi et al. [31], starting from an acrylate resin with nickel particles. Some studies also focused on particle or fiber reinforcements with field-assisted stereolithography technologies to manipulate the fillers inside the liquid resin during the curing process [32].

Magnetic particle-reinforced composite found several applications in different industrial sectors, among them the electrical and medical industries [33]: different literature studies analyze the addition of magnetite (Fe_3_O_4_) [34,35] and strontium ferrite (SrFe_12_O_19_) particles, showing a good feasibility of the production process [29]. On the other hand, the benefits of short fiber reinforcements [21] on additive manufacturing processes have also been investigated in the literature. Sano et al. [36] investigated the influence of glass powder, short fiber, and fabric on the stereolithography manufacturing of clear resin, showing an increment in the mechanical properties in terms of tensile strength and Young’s modulus. Dong et al. [37] utilized chopped quartz fiber-reinforced fused silica composite in the stereolithography process, successfully achieving a sintered structure without defects.

Nano-size reinforcement applied to addictive manufacturing processes has also drawn academic interest, and some studies can be found [38] together with industrial application study cases [39]. In this regard, Dos Santos et al. [40] investigated the mechanical behavior of photocurable epoxy acrylate resin with multiwalled carbon nanotubes (MWCNT), discovering that the manufactured specimens had an increment in their stiffness and hardness. Thermal characterizations of photopolymer resin with different nanofillers used for SLA manufacturing have also been conducted by Hu et al. [41].

Considering the literature background presented so far, this research aims to unfold the potentials and limitations of the particle-reinforced SLA process based on a methacrylate resin filled with an average size of 1 μm tungsten (W) particles with two wt. concentrations equal to 4% and 10%, respectively. Tungsten metal powder reinforcement has not been investigated yet in stereolithography manufacturing systems and, furthermore, tungsten has several attractive properties such as high tensile resistance [42]. Moreover, the use of bigger particle dimensions can hamper the strict dimensional and surface quality of the final product considering the precise and sensitive process of stereolithography. To assess the effect of the W particles’ addition to the base materials, tensile, compressive, and impact specimens with unfilled methacrylate resin, 4% wt. and 10% wt. W particles were prepared and evaluated. Furthermore, to better understand the reciprocal interaction between the methacrylate resin base resin and the W particles reinforcement, Field Emission Scanning Electron Microscope (FE-SEM) analyses were conducted on the failure and fracture area of unfilled and reinforced specimens carved from tensile and impact samples.

## 2. Materials and Methods

### 2.1. W-Particle-Reinforced Methacrylate Composite Preparation

**Figure 1:**
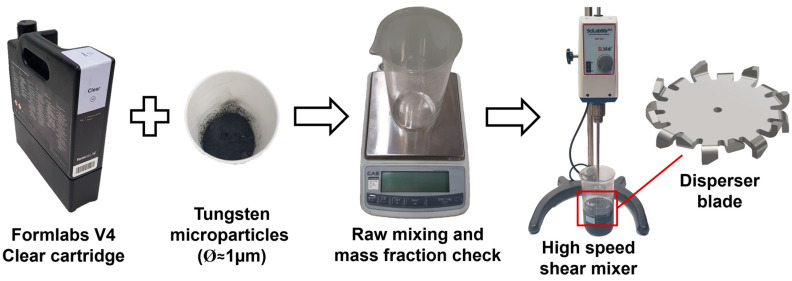
Production process of W-particle-reinforced methacrylate composite starting with Formlabs V4 clear resin and adding micro-size W particles.

The thermoset resin used to realize the unfilled specimens and as the base for the W-reinforced material is the Formlabs Clear resin V4, a methacrylate photopolymer. For the creation of the particle-reinforced material, Avention tungsten powder, with a powder mean size of 1 µm and a purity level of 99.9%, was used (Figure 1). The mass of the tungsten particles and methacrylate resin were measured by the CUX4200HX electronic scale (CAS, Yangju, Republic of Korea) with a precision of 0.01 g. The mixing operation begins by pouring 1/4 of the resin into a graded baker, followed by 1/3 of the particles’ reinforcement, and so on. This stacking approach allows for only resin at the bottom and top of the mixing baker and avoids particle dispersion in the working environment or dry regions at the bottom. Afterward, to obtain a uniform composite and avoid phase separation or clustering, the raw compound was subjected to a high-speed mixing process by employing the SMS-50A SciLabStir stirrer (Jung-Il Science Co. Ltd., Hwaseong, Republic of Korea) with a customized shear disperser for 2 h. As shown in Figure 1, the disperser design presents inclined blades at the top and bottom to create a highly turbulent flow above and beneath the rotation center.

After the mixing operation, the composite resin was poured into the original Formlabs Clear cartridge, previously cleaned with isopropyl alcohol (99.9% concentration) to avoid contamination with unfilled resin, thus a reduction in the W particle concentration was possible. For the preparation of the 4% and 10% mass fraction W-particle-reinforced methacrylate composite, two different cartridges were employed to avoid contamination throughout all manufacturing stages, including the tank installed in the Form3+ (Formlabs, Boston, MA, USA) SLA machine.

As concerns the mixing operation, it should be noted that due to the higher density of the W particles (~19.3 g/cm^3^) with respect to the methacrylate resin (~1.15 g/cm^3^), phase separation starts to become evident after 48 h and is almost complete after 72 h from the end of the high-speed mixing operation. Accordingly, the resin should not be left to rest in the same position for more than 24 h to avoid phase separation. Moreover, preliminary tests on the maximum allowable tungsten percentage on methacrylate were conducted, showing that a concentration higher than 10% Tungsten particle reinforcement led to production problems such as specimens that were porous and had poor dimensional accuracy. Selecting the first mass fraction percentage (10%), it was observed that preliminary tests with lower concentrations of 4% Tungsten particles inside the methacrylate matrix did not lead to any relevant mechanical improvement.

### 2.2. Specimens’ Specifications and Manufacturing

**Figure 2:**
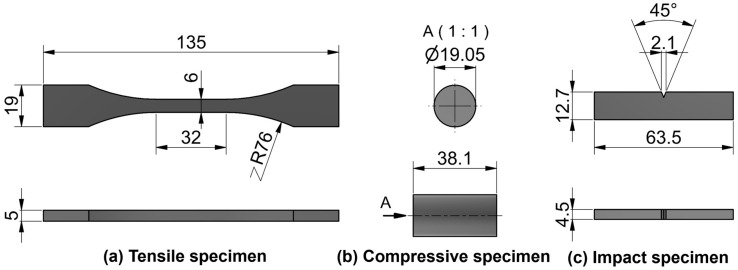
Specimens’ dimensions designed with Autodesk Inventor. (**a**) Tensile specimen shaped in accordance with ASTM D638, (**b**) compressive specimen complying with ASTM D695, (**c**) impact specimen according to ASTM D256.

**Figure 3:**
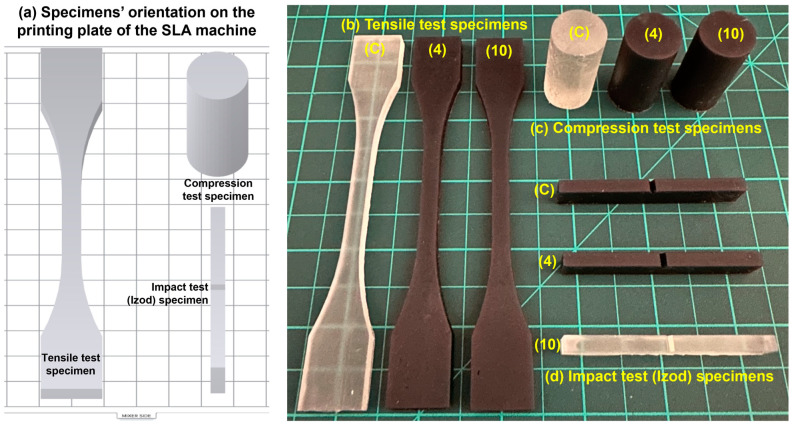
(**a**) Specimens’ orientation on the printing plate for tensile, compressive, and impact tests. Manufactured (**b**) tensile, (**c**) compression, and (**d**) impact specimens with unfilled methacrylate resin (C), 4% W-particle-reinforced methacrylate composite (4), and 10% W-particle-reinforced methacrylate composite (10).

To characterize the W-particle-reinforced methacrylate composite and benchmark its performances with respect to the unfilled V4 Formlabs Clear resin (Formlabs, Boston, MA, USA), tensile, compressive, and impact specimens were designed and manufactured, as shown in Figure 2. The tensile specimens, in Figure 2a, were based on the ASTM D638 Type II [43], preferred to Type I due to the fragility of the unfilled resin, which showed failure initiation close to the jigs’ region in some of the specimens. The compressive specimens, in Figure 2b, were designed according to the ASTM D695 standard [44] with a 2:1 height-to-diameter ratio. Finally, for the impact specimen, in Figure 2c, the geometry and testing conditions reported in ASTM D256 [45] were considered since the tests were conducted with the Izod configuration. The same specimens’ dimensions in Figure 2 were considered for manufacturing unfilled, 4% wt., and 8% wt. specimens, as shown in Figure 3.

For the manufacturing of the specimens, the Form3+ low force stereolithography (LF-SLA) printer was employed with a layer resolution of 0.025 mm, subsequently washed for 15 min in an ultrasonic bath with isopropyl alcohol (99.9% concentration) and left to dry at room temperature for 2 h. Since all three specimens have at least one flat surface and no undercuts, samples were printed directly in the building plate without any support to increase the surface quality and decrease the production time. Moreover, each specimen was printed with the same printing layout, to avoid any effect of the print direction on the material behavior. The impact specimens were printed with a layer resolution of 0.025 mm as per the other mechanical test samples. Accordingly, no further sharpening was carried out before the impact test, since the shape of the notch resulting from the printing phase was correct as recommended by ASTM D256 standards.

Regarding the curing operation, in light of a previous study by one of the authors, a temperature of 60 °C for 80 min was employed for all the specimens. After completing the curing, the specimens were left to cool down at room temperature for 2 h and then sealed in plastic bags until the relevant tests. 

Before each of the three characterization experiments, the key dimensions of each specimen were measured by a CD-15APX caliber (Mitutoyo, Sakado, Japan) with a precision of 0.01 mm, and their weight was checked with a CUX4200HX electronic scale (CAS, Yangju, Republic of Korea). Each specimen was manufactured and tested five times to verify the consistency of the manufacturing process. The mean values and standard deviations for the key dimensions and the specimens’ weights are reported in Section 3 of the paper.

### 2.3. Specimens’ Testing Procedures

**Figure 4:**
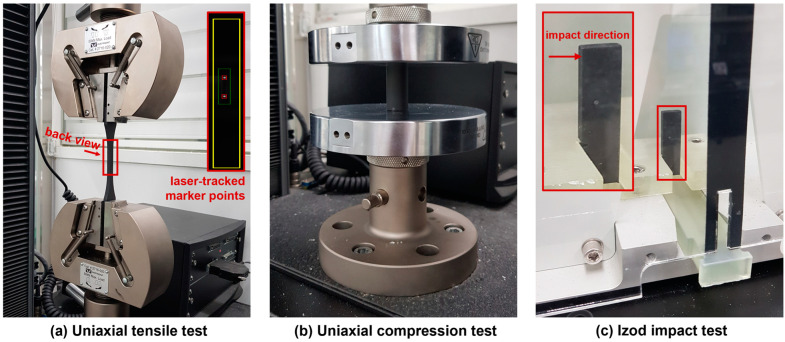
Mechanical tests employed to characterize the W-particle-reinforced methacrylate composite and the Formlabs V4 Clear resin. (**a**) Static uniaxial tensile test and (**b**) static uniaxial compression test with Instron 5969 Dual Column Testing system. (**c**) Izod impact test with Tecquipment TE15 system.

To assess the mechanical performances of the W-particle-reinforced methacrylate composite, three tests were conducted: uniaxial tensile, Figure 4a, uniaxial compression, Figure 4b, and an Izod impact test, Figure 4c.

For the tensile and compressive test, the Instron 5969 Dual Column Testing system was used with different sets of jigs. For the tensile experiments, the Instron AVE2 (Instron, Norwood, MA, USA) laser strain gauge extensometer was employed, as shown in the detail of Figure 4a. The two points are marked on the specimen’s surface at a ~25 mm distance, symmetrically from the center, identified and locked before the test, and their position was tracked throughout the experiment to acquire an accurate measurement of the displacement. For the compression tests in Figure 4b, the vertical displacement was measured by the Instron 5969 machine (Instron, Norwood, MA, USA) columns’ encoders. Finally, for both tensile and compression experiments, the load was measured by the Instron 2580 loadcell (Instron, Norwood, MA, USA) with a maximum capacity of 50 kN and a resolution of 1/1000. For the tensile tests, the vertical movement of the jig was set to 2.5 mm/min, whereas it was set to 2.0 mm/min for the compression tests.

Concerning the Izod impact test, carried out by employing the Tecquipment TE15 system (TecQuipment Ltd., Nottingham, UK), the V-notch specimens were installed within the machine chamber as shown in Figure 4c and impacted with a hammer having an initial potential energy of 2.34 J. The impact energy, recorded after the test, is then divided by the relative sample’s cross-section to estimate the impact strength. The engineering stress–strain curves for tensile and compressive loading conditions and the impact strength results for unfilled, 4% wt., and 8% wt. W-reinforced methacrylate composite specimens are all summarized in Section 3.

## 3. Results and Discussion

### 3.1. Tensile Properties

**Figure 5:**
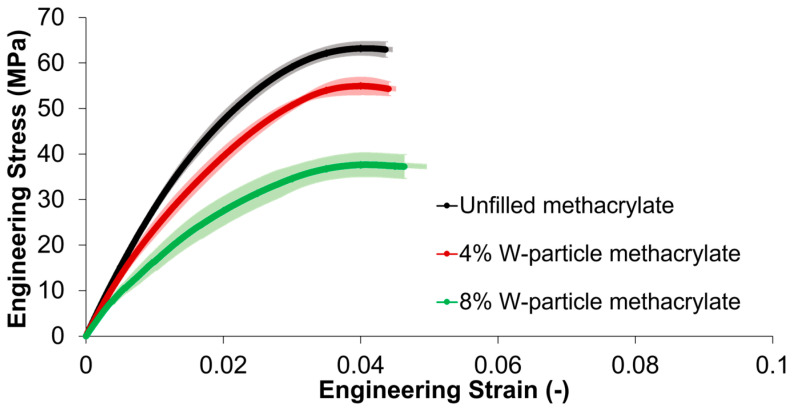
Engineering tensile stress–strain curves with standard deviations for unfilled methacrylate resin, 4%, and 8% W-particle-reinforced methacrylate composite.

Concerning the uniaxial tensile tests on unfilled methacrylate, 4% and 10% W-particle-reinforced composite, the results are summarized in Figure 5, where a quasi-brittle stress–strain relationship is identified with a drop in elastic modulus and ultimate tensile strength (UTS) as W particle concentration increases.

First of all, the behavior of all the three materials, both unfilled and composite, is defined as quasi-brittle since both an elastic and a slight hardening behavior before failure were identified before the final failure. The standard deviations highlighted in Figure 5 show that for the unfilled and 4% W-particle methacrylate composites the results data are clustered tightly around the average value, while for 4% W-particle methacrylate composite data are slightly more scattered. For the tensile behavior, the unfilled methacrylate polymer shows an elastic modulus of 3.1 GPa and a UTS of 62.9 MPa, whereas the addition of 4% of tungsten particles reduces them by 11.1% and 13.7%, and by reaching 10% W particles results in a further reduction down to 34.8% and 40.8%, respectively. The progressive addition of W particles results in a slight increase in the material ductility, calculated as percent elongation until failure for the tensile specimens. For the unfilled samples, the average ductility value is 4.36, while for the 4% W-particle methacrylate composite it is 4.41 (+1.2%), and for 10% W-particle methacrylate composite it is 4.63 (+6.3%), both with respect to the unfilled methacrylate. The reduction in the tensile behavior as a consequence of the introduction and increase in W particle reinforcement percentage is explainable by two factors. First, although the tungsten material properties are far superior to those of the matrix material, due to their aspect ratio close to 1, the microparticles do not promote any significant shear stress transfer with the matrix. Second, as will also be shown in Section 3.4, the boundary between the methacrylate matrix and W particles appears to be weak in nature, creating fracture initiation points rather than reinforcing the base polymer [25]. Both these phenomena result in a reduction in the tensile modulus and UTS with a slight increase in the tensile failure strain, as shown in Figure 5.

SEM micrographs reported in following Section 3.4 help with analyzing the interaction between tungsten particles and methacrylate resin, showing a poor adhesion between the two of them. For the case of the tensile test, the applied loading condition makes the poor adhesion between the tungsten particles and methacrylate matrix to be both a crack initiation spot and also a weakening of the cross-section. Specifically, in the case of the unfilled methacrylate, the applied load is redistributed across the whole cross-section of the tensile specimen. On the other hand, in the case of the tungsten-reinforced specimens, the boundary between matrix and particles results in a local stress concentration and in a reduction in the effective cross-section of the specimen.

### 3.2. Compressive Properties

The mechanical behavior displayed by both unfilled and composite materials differs significantly between tensile and compressive loading conditions.

**Figure 6:**
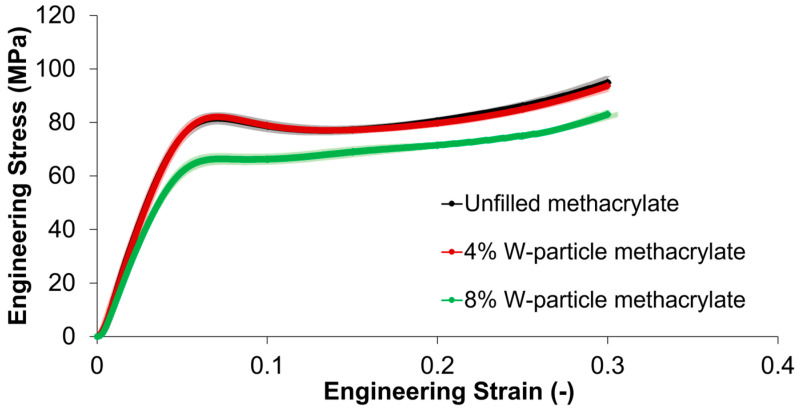
Engineering compressive stress–strain curves with standard deviations for unfilled methacrylate resin, and 4% and 8% W-particle-reinforced methacrylate composite.

Concerning the compressive behavior displayed in Figure 6, it can be discerned that its elastic component is approximately two times that of the tensile one, consistent with the nature of the methacrylate matrix. Regarding the standard deviations, each material analyzed in the compressive test shows that data are well clustered around the calculated average value. Moreover, there is no significant difference between unfilled methacrylate and the 4% W-particle methacrylate composite, meaning that low percentages of tungsten reinforcement do not affect compressive behavior. On the other hand, concerning the 10% W-particle methacrylate composite, it can be seen that the behavior and that a higher concentration results in a lower stress peak before the softening region, which is also less pronounced. The compressive elastic modulus for unfilled resin and 4% tungsten particles in the thermoset matrix is respectively, 1.6 GPa and 1.5 GPa (−2.6%), while the compressive yield strength YS is 120 MPa and 122.1 MPa (+1.7%). Compared to the 10% tungsten particles in the thermoset matrix, the reduction is 16.3% and −1.4%, respectively. Regarding compressive failure behavior, all specimens show instability arising from 0.3 strain with an abrupt failure between 0.3 and 0.35.

In general terms, the addition of 4% W particles has little to no effect on the compressive behavior, whereas the higher concentration of 10% results in a flattening of the softening peak in the 0.05 ≤ ε ≤ 0.1 engineering strain range (Figure 6). In addition, by comparing the stress–strain curves of Figure 5 and Figure 6, unfilled and reinforced methacrylate composites have higher stiffness and ultimate strength under compressive loading conditions than tensile ones.

### 3.3. Impact Properties

**Figure 7:**
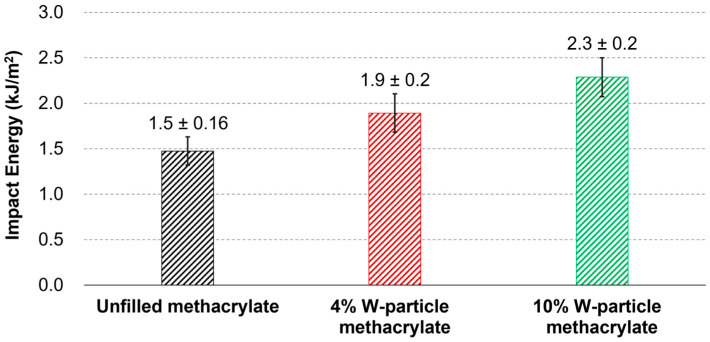
Impact energy (kJ/mm^2^) for unfilled methacrylate and W-particle-reinforced materials having 4% and 10% concentrations.

Contrary to what is observed for the case of tensile and compressive properties, as reported in Section 3.1 and Section 3.2, the impact strength experiences a strong improvement thanks to the addition of W particles, as reported in Figure 7. The impact energy measured during the tests was divided by the measured specimens’ cross-sections.

The average values measured over five specimens for the impact strength show 1.475 kJ/m^2^ for the unfilled methacrylate polymer, 1.893 kJ/m^2^ for the 4% concentration, and 2.287 kJ/m^2^ for the 10% W particles concentration, respectively. The improvements, with respect to the unfilled resin, are quantified in 28.3% and 55.1%, showing the positive effect of the W particles on the impact strength. As mentioned in Section 3.1 and as shown in SEM micrographs reported in following Section 3.4, the relatively low joining strength between the methacrylate matrix and the tungsten reinforcement results in an obstacle for fracture propagation, absorbing more energy during the impact test. The reason for this is given by the fact that the path of minimum fracture energy goes around the particles instead of through them, regardless of the adhesion strength between the particles themselves and the polymer matrix. On top of that, as will be shown in Section 3.4, the fracture surface of the impact specimens shows a clean surface on the top of the exposed W particles, proving that the separation occurs on the particles–matrix interface rather than within the matrix itself.

### 3.4. Failure and Fracture Surface SEM Analysis

**Figure 8:**
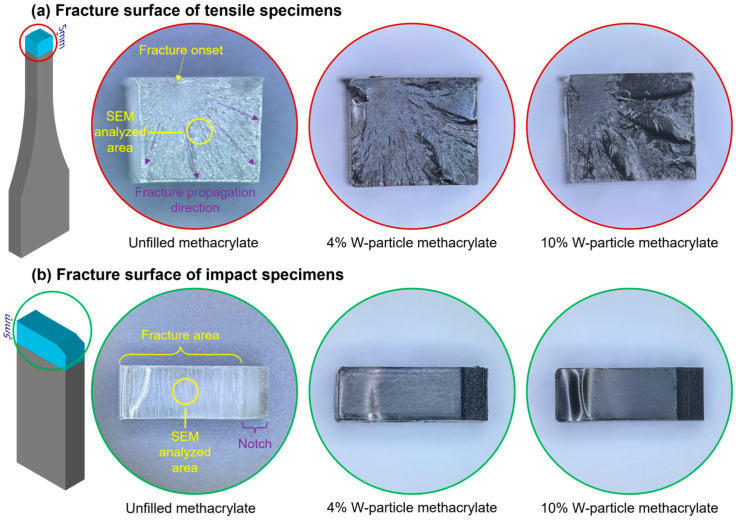
Cut samples for SEM analysis. (**a**) Failure surface of the tensile specimens with details on the failure onset and propagation direction and SEM-analyzed area position. (**b**) Fracture surface of the impact specimens with details on the in-built notch and fracture area consequent to the impact and SEM-analyzed area position.

Field emission scanning electron microscope FE-SEM analyses were conducted with the JSM-7100F system (JEOL Inc., Peabody, MA, USA) on the failure and fracture surface of tensile and impact specimens, Figure 8. Considering the abrupt and random nature of the failure, no FE-SEM analyses were conducted on compression specimens. From the two halves of the tensile and impact specimens, 6 × 5 × 5 mm and 12.7 × 4.5 × 5 samples were carved and coated with a Pt layer to enhance the contrast during the SEM analysis.

**Figure 9:**
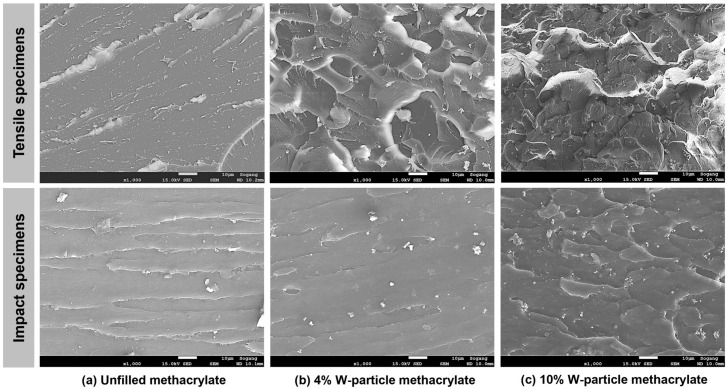
SEM images of the failure surfaces for tensile and of fracture propagation area for impact specimens for (**a**) unfilled methacrylate, (**b**) 4% W-particle-reinforced methacrylate composite, and (**c**) 10% W-particle-reinforced methacrylate composite.

Considering the six specimens presented in Figure 8, the surface patterns of the failure propagation region for the tensile specimens and those on the fracture areas for the impact one are reported in Figure 9, where all pictures are taken with a 1000× magnification.

Regarding the unfilled resin, the failure surface of the tensile specimen is clean with partial fibrillation, compatible with the failure behavior of a quasi-brittle material under tensile loading conditions [17]. An analogous mention can be made to the unfilled fracture surface of the impact specimen, where it can be seen as an almost clear fracture surface, denoting again a quasi-brittle material, even if both mechanical tests, tensile and impact, are different. On the other hand, the higher the W particle content becomes, the higher the roughness of the failure and fracture surface, especially in the case of tensile specimens. From this first analysis, it is clear that the W particles act as an obstacle to fracture propagation during the impact test, which, in turn, results in a higher roughness for the fracture surface.

**Figure 10:**
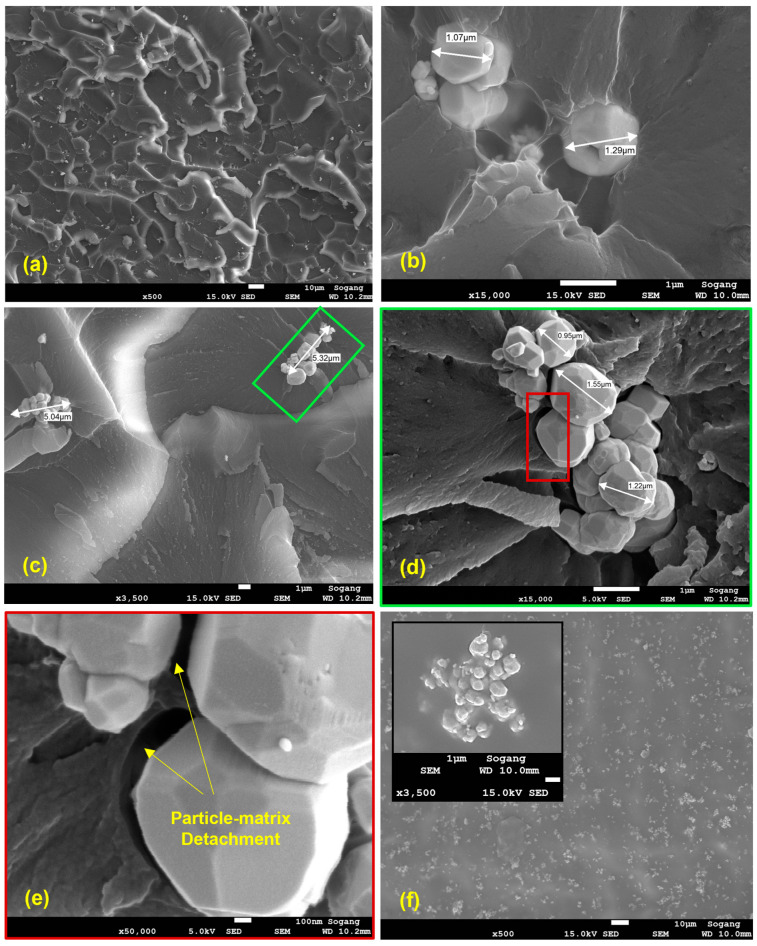
(**a**) Failure surface of a 4% W-particle tensile specimen with (**b**) details of single particle’s dimensions, (**c**,**d**) clusters’ dimensions, and (**e**) example of methacrylate matrix—W particle detachment on the failure surface. (**f**) Fracture surface of 8% impact specimen with detail of cluster embedding in the matrix. The green box in (**c**) is magnified in (**d**) whereas the red box in (**d**) is magnified in (**e**), respectively.

In Figure 10 the W particle clusters are visible as white elements on the fracture surfaces of the impact specimens and on that of the 4% concentration tensile specimen, whereas for the 10% concentration tensile specimen, the high surface roughness makes it harder to spot the W particles. To better highlight the intertwining between the methacrylate matrix and the W-particle reinforcement, Figure 10 shows an overview of a failure and a fracture surface and various magnified pictures of single particles and clusters from the FE-SEM scans of the failure surface of tensile specimens.

The failure surface of a tensile specimen with 4% W particles in Figure 10a, presents a relatively uniform pattern of exposed tungsten particles (Figure 10b,c) and clusters with an average length of ~5 μm (Figure 10b), and with an average particle size of ~1 μm. Considering the high number of W particle clusters exposed on the separation surface, the failure propagation following the onset of Figure 8 most likely arises at the boundary between the reinforcement and the matrix (Figure 10d,e).

This fact also results in some localized pull-out, or detachment, of the W particles from the methacrylate matrix, as shown in the magnification of Figure 10e. The fact that the particle–matrix joining strength is lower than the methacrylate UTS results in a reduction in both the elastic modulus and strength, and in the progressive increase in the failure strain, as shown in Figure 5 and discussed in Section 3.2 of the tensile results.

With the same rationale but different results, the presence of the W particles creates an obstacle for fracture propagation during the impact test. For this second test, the load is applied perpendicularly to the fracture surface, and as shown in Figure 10f, the fracture surface of the impact specimens shows most of the tungsten mostly embedded within the methacrylate matrix rather than exposed, as it was in the case of the tensile specimens (Figure 10a–e). For the case of the unfilled impact specimen, the fracture surface is free to propagate without obstacles; on the other hand, when the W particle reinforcement is added, it creates an obstacle to the propagation, making the fracture surface adjust its trajectory to avoid the reinforcement. This fact results in an increase in the energy required for creating a new fracture surface, increasing the impact strength (Figure 7), thus resulting in a higher effectiveness of the reinforcement.

**Figure 11:**
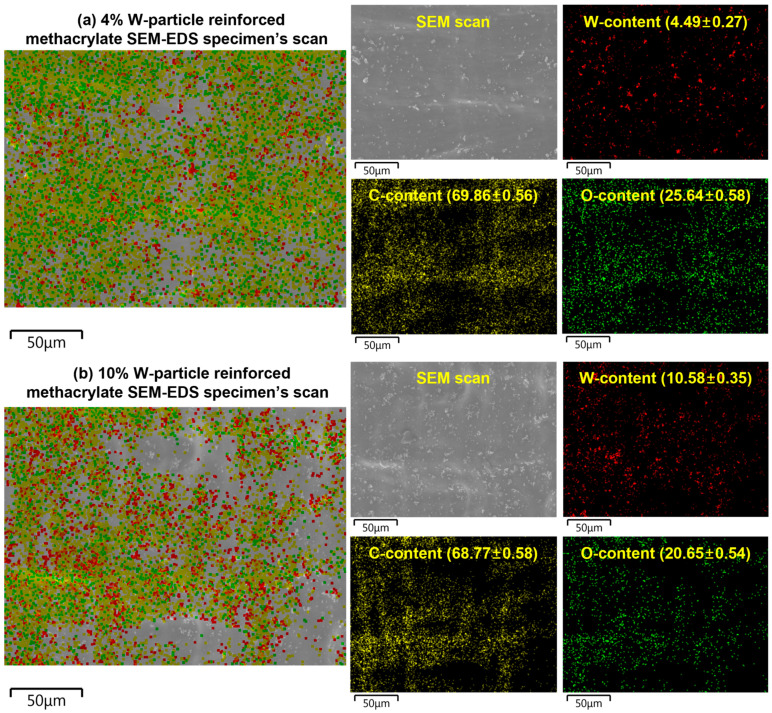
SEM-EDS analysis of the fracture surface of the W-particle-reinforced methacrylate composite impact specimens for (**a**) 4% and (**b**) 10% concentrations. The values between brackets are the percentage concentrations and standard deviations.

Finally, to verify the homogeneity of the W particle reinforcement within the methacrylate matrix, the smooth surface of the impact specimens was subjected to Energy Dispersive Spectroscopy (EDS). This analysis maps the distribution of the elements on the tested samples, allowing for visual and quantitative estimation of the W particle reinforcement distribution, as shown in Figure 11. In Figure 11, Oxygen (O) and Carbon (C) elements are the base elements of the methacrylate matrix. First, the regions of the sample where W-particles seem to be missing are, in fact, areas where the reinforcement is embedded too deep in the methacrylate matrix to be detected by the EDS. On the other hand, in the remaining regions, the W particle reinforcement looks uniformly distributed even though clusters are scattered throughout the sample.

As shown in Figure 11, both weight percentages are remarkably similar to the theoretical one designed during the creation of the W-particle-reinforced materials, showing the reliability of the implemented manufacturing process. Interestingly, the clustering does not change significantly between the 4% and 10% wt., suggesting that even higher concentrations might be a feasible solution, even though the effect on the elastic and yield properties might be too severe to justify it.

### 3.5. Dimensional Accuracy

**Figure 12:**
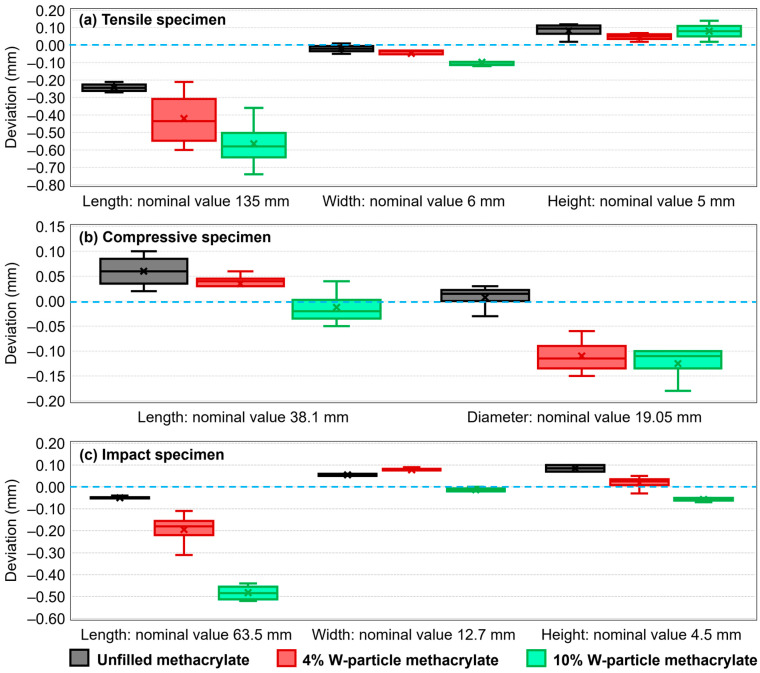
Dimensional deviation for the (**a**) tensile specimen, (**b**) compressive specimen, and (**c**) impact specimen for unfilled and W-particle-reinforced (4% and 10%) resin.

Considering the peculiar nature of the particle-reinforced SLA process, the dimensions of the manufactured specimens were verified before each test and compared with the theoretical (CAD) values, as reported in Figure 12. All dimensions have been verified with a digital caliper with a 0.01 mm accuracy and after the post-curing process.

Generally speaking, the printing detail level is uniform and consistent, while the dimensional accuracy slightly decreases with a higher percentage of W particles. Although remarkably small in size, the presence of reinforcement particles creates an irregularity in the methacrylate matrix. In addition, all specimens have been manufactured directly on the printing plate (Figure 3), a fact that makes the layer-controlled direction more precise than the other two planar ones, as testified by the comparison between Figure 12b with Figure 12a,c. In addition, the Formlabs Form3+ printer is not designed to allow for customized printing, meaning that the intensity of the curing beam as well as exposition time during the manufacturing process cannot be adjusted. To this aim, a viable solution to be explored in further research is to employ other cartridge chips belonging to a different material to enhance the range of practical options for the preliminary photopolymerization energy provided during the manufacturing.

## 4. Conclusions

This research analyzed the SLA manufacturing process and the mechanical responses under tensile, compressive, and impact loading conditions of a methacrylate photopolymer resin filled with 4% and 10% wt. of tungsten micro-size particles. The main conclusions and considerations of this work are summarized as follows:

i The manufacturing process developed successfully achieved the production of specimens with two different percentages of tungsten micro-size particle reinforcements (4% and 10%). Three different samples have been produced in different steps, assuring the repeatability of the process.
ii The tensile material properties of 4% and 10% tungsten-particle-reinforced specimens showed a decrease in the elastic modulus (−11.1% and −34.8%, respectively) and ultimate tensile strength (−13.7% and −40.8%, respectively), while a slight increase in the failure strain (+1.1% and 6.1%, respectively) compared to the unfilled material properties. On the other hand, 4% tungsten reinforcement shows a similar behavior to the unfilled specimens under compression load, with an elastic modulus of 1.5 GPa and 122 MPa of UTS. The 10% tungsten-reinforced composite showed a decrease in the compressive elastic modulus (−16.3%) and UTS (−1.4%) compared to the unfilled material.
iii Concerning the impact tests, an increment in the tungsten particles inside the resin matrix led to a strong increment in the impact strength. Compared to the unfilled material samples, the 4% tungsten-particle-reinforced specimens show an average increment of 28.3% in the impact strength, while 55.1% for the 10% tungsten-particle-reinforced material.
iv The SEM revealed the interaction between the microparticle and the resin matrix, showing an average particle size of 1.2 µm and a cluster maximum envelope of 5.3 µm. The polymer matrix is well distributed throughout the failure and fracture surfaces, while the tungsten particles shows partial debonding and low adhesion strength with methacrylate matric, justifying the decrease in the tensile elastic modulus and UTS.
v The obtained mechanical characteristics of the tungsten-particle-reinforced composite, namely higher impact strength with some compromise in the UTS and elastic modulus can be helpful in various automotive, sports equipment, and protective gear devices where impact resistance is directly linked to the functionality of the manufacturing component.
vi Future work should be oriented toward optimizing the production process with customized process settings to further reduce cluster dimensions and enhance cohesion between the tungsten particles and the resin material.

## Data Availability

The research data related to this article are made available on request to the corresponding author.
